# The association between serum uric acid levels and the cardiometabolic phenotype among healthcare workers of Tabriz University of Medical Sciences

**DOI:** 10.34172/jcvtr.32902

**Published:** 2025-03-18

**Authors:** Mohammadhossein Somi, Negin Frounchi, Seyed Sina Zakavi, Alireza Ostadrahimi, Neda Gilani, Elnaz Faramarzi, Sarvin Sanaie

**Affiliations:** ^1^Liver and Gastrointestinal Diseases Research Center of Tabriz University of Medical Sciences, Tabriz, Iran; ^2^Student Research Committee, Tabriz University of Medical Sciences, Tabriz, Iran; ^3^Nutrition Research Center, Tabriz University of Medical Sciences, Tabriz, Iran; ^4^Department of Statistics and Epidemiology, Faculty of Health, Tabriz University of Medical Sciences, Tabriz, Iran; ^5^Neurosciences Research Center, Tabriz University of Medical Sciences, Tabriz, Iran

**Keywords:** Abdominal obesity, Uric acid, Metabolic syndrome, Body mass index

## Abstract

**Introduction:**

It is unclear whether hyperuricemia can be considered as an independent risk factor or just as a marker to represent the correlation between uric acid levels and other risk factors of MetS. In this work, we intend to study the correlation between serum uric acid (SUA) and the cardiometabolic phenotype among Tabriz University of Medical Science healthcare workers.

**Methods:**

In this cross-sectional study, anthropometric measurements, serum fasting blood sugar (FBS), triglyceride (TG), cholesterol, high-density lipoprotein (HDL), liver enzymes, blood urea nitrogen (BUN), SUA, creatinine (Cr), and blood pressures of 1,451 healthcare workers were evaluated. MetS was diagnosed based on ATP III. We classified the participants into four cardiometabolic phenotypes: metabolically-healthy lean (MHL), metabolically-unhealthy lean (MUHL), metabolically-healthy obese (MHO), and metabolically-unhealthy obese (MUHO).

**Results:**

MHL (26.6%) and MHO (65.8 %) had the highest prevalence rates in the first and second SUA categories, respectively (*P*≤0.001). Compared to the lowest SUA category, the odds of MHO and MUHO increased by 3.13 (95% CI 2.21–4.44) and 5.50 (95%CI 3.53–8.57) in the highest category, respectively. This trend was not observed regarding the association between MUHL and the SUA classification.

**Conclusion:**

We propose using the easily-measured SUA level as a marker for early diagnosis of at-risk MUHL and MHO individuals to administer proper interventions. Further prospective studies are needed to identify the effects of SUA on the progression of MetS in various body-size subgroups.

## Introduction

 One of the growing health challenges of the twenty-first century is metabolic syndrome (MetS), the prevalence of which is on the rise in both developed and developing countries.^1^ Global prevalence of MetS ranges from 12.5 to 31.4%,^1^ while based on various definition criteria, 32.1 to 47.6 percent of the Iranian population suffer from MetS.^2^ The set of risk factors for cardiovascular diseases and type 2 diabetes mellitus that often coexist rather than happen coincidentally is known as the metabolic syndrome.^3^ MetS can predict the risk of developing diabetes, cardiovascular diseases,^4^ and cancer in people.^5^ However, understanding the pathogenesis of MetS, including its underlying mechanisms, the development stages, and the interactions among its individual components, is a very complex task. ^6^

 Metabolic syndrome has several risk factors, including hypertriglyceridemia, central obesity, hypertension, and insulin resistance, which can be accompanied by increased uric acid levels, i.e., a pro-inflammatory agent that affects endothelial cells.^7^

 Several studies have reported a relationship between urate and MetS, as well as its components.^8-11^ A correlation between BMI and circulating urate concentrations has also been reported. Individuals classified in the high BMI category show high uric acid levels, strongly associated with MetS.^12,13^ The genetic predisposition of people with high uric acid levels is associated with blood pressure elevation and dyslipidemia, but not with obesity and diabetes, all of which are components of MetS. This may suggest that high serum uric acid (SUA) levels may be related to a separate, obesity-independent pathway for the development of MetS.^14^

 The exact metabolic biomarkers that show healthy individuals’ likelihood of becoming metabolically unhealthy during their lifetime are not fully understood. We hypothesize that elevated SUA levels may play a role in the pathogenesis of MetS in metabolically-unhealthy lean (MUHL) or metabolically-unhealthy obese (MUHO) individuals. As of now, it is unclear whether hyperuricemia can be considered an independent risk factor or just as a marker to represent the correlation between uric acid levels and other risk factors of MetS. To our knowledge, no other work has focused on the relationship between SUA and cardiometabolic phenotypes. In this work, we studied the correlation between SUA and the cardiometabolic phenotypes of healthcare workers of Tabriz University of Medical Sciences.

## Materials and Methods

 In this cross-sectional study, we used the data of a Healthcare worker cohort that is part of a large prospective epidemiological study in Iran (the Persian Cohort Study).^15^ The healthcare worker cohort study was carried out on healthcare workers in 2019 as a part of the Azar Cohort Study, conducted by the Liver and Gastrointestinal Diseases Research Center of Tabriz University of Medical Sciences.^16^ This cohort study aimed to evaluate individuals linked with Tabriz University of Medical Sciences (TBZMED), including healthcare employees in hospitals, schools, and district health networks. This study intended to identify the risk factors of non-communicable diseases (NCD) among TBZMED healthcare professionals, employees, and professors.

 Our baseline assessment consisted of a face-to-face health interview or a health examination regarding a broad range of established and novel risk factors for NCDs.

 Data from a total of 1,458 participants were used for this study. This is a cross- sectional study and data of the cohort study was used, we included all participants who enrolled from January 2019 to December 2019.All involved participants provided written informed consent, and the study was approved by the Ethics Committee of Tabriz University of Medical Sciences (IR.TBZMED.REC.1400.657).

 Inclusion criteria included full-time and long-term contract employees aged 18 to 65 years and particiapnts who were pregnant or breastfeeding and were planning to retire within the next five years excluded from the study.

###  Demographic Characteristics 

 We used a questionnaire to evaluate the demographic characteristics, including age, gender, marital status, and educational level. Moreover, the questionnaire assessed lifestyle patterns, i.e., smoking, drug use, hookah use, alcohol consumption, and passive smoking.

###  Anthropometric and Blood Pressure Measurements 

 All subjects’ body weight, height, and waist circumference were measured, and their body mass index (BMI) was determined using the standard formula of weight (kg) divided by heightsquared(m^2^). The anthropometric measurements are described in detail elsewhere. Blood pressure was measured while the participant was seated after ten minutes of rest. Measurements were made by a trained nurse twice on each arm across a two-minute interval using a mercury sphygmomanometer (Rudolf Richter, DE-72417, Germany). The average values were calculated and used in the analysis as the systolic and diastolic blood pressures.

###  Biochemical Factors

 After an overnight fast (12 hours), blood samples were obtained from the participants to determine serum uric acid (SUA), fasting blood sugar (FBS), triglyceride (TG), high-density lipoprotein (HDL), cholesterol, aspartate aminotransferase (AST), alanine aminotransfer (ALT), alkaline phosphatase (ALP), gamma-glutamyl transferase (GGT), blood urea nitrogen (BUN), and creatinine (Cr) using enzymatic methods. In addition, low-density lipoprotein (LDL) levels were calculated based on the Friedewald formula.

###  Definition of MetS

 The Adult Treatment Panel III (ATP III) of the National Cholesterol Education Program defines individuals with MetS as subjects who meet three or more of the following conditions: hypertension, defined as a systolic blood pressure ≥ 130 and/or a diastolic blood pressure ≥ 85 mmHg or use of antihypertensive medications; waist circumference ≥ 102 cm in men and ≥ 88 cm in women; hypertriglyceridemia, defined as TG ≥ 150 mg/dl or treatment for elevated triglycerides; HDL-C values < 40 mg/dl in men and < 50 mg/dl in women; and high fasting glucose ( ≥ 100 mg/dl) or the use of glucose-lowering medications.^3^

 In this study, subjects were categorized into four cardiometabolic phenotypes based on the BMI cutoff point of 25 kg/m^2^ and the presence or absence of MetS: MUHO, metabolically-unhealthy obese (presence of MetS and BMI ≥ 25 kg/m^2^); MUHL, metabolically-unhealthy lean (presence of MetS and BMI < 25 kg/m^2^); MHO, metabolically-healthy obese (no MetS and BMI ≥ 25 kg/m^2^); and MHL, metabolically-healthy lean (no MetS and BMI < 25 kg/m^2^).^17^

###  Statistical Analysis

 Statistical analysis was performed using IBM SPSS Statistics version 16 (IBM, Chicago, IL). The normality of data was evaluated by Skewness and Kurtosis indices and Q-Q plot. Continuous variables with normal distribution were expressed as mean ± standard deviation (SD), with analytic analysis performed using the Independent Samples T-Test. Variables without normal distribution were presented as median with interquartile ranges as well as mean ± SD, and statistically compared using the Mann-Whitney U test. Categorical variables were presented as numbers (with percentages). The Chi-square statistical test was used for categorical data.

 The participants were categorized into two groups based on SUA levels, with the first category for SUA levels less than 5 mg/dl and the second category over 5 mg/dl.

 In addition, multinomial logistic regression analysis was performed to determine the relationship between the cardiometabolic phenotype (MHL,MUHL,MHO,MUHO) and the SUA classification. Crude and adjusted (adjusted for age, gender, educational level, and current smoking status) odds ratios (OR) were calculated along with their corresponding 95% confidence intervals (95%CIs). In this study, MHL was used as a reference group.

 We considered BMI the basis for classification, so seven underweight participants were excluded. Eventually, statistical analysis was carried out on 1,451 subjects. Moreover, P-values below 0.05 were considered statistically significant.

## Results

 Of 1451 particiapnts, 726 (50%) were females and the mean age (years) of participants was 42.53 ± 6.72. Table 1 presents the baseline characteristics of the participants based on the SUA classification. The second category included a higher percentage of male and married participants than the first one (*P* < 0.001). Moreover, the MHL (26.6%) and MHO (65.8%) groups had the highest prevalence in the first and second SUA categories, respectively (*P* < 0.001). We observed an increasing trend in the mean values of WC, TG, cholesterol, FBS, BUN, Cr, SBP, DBP, BMI, and liver enzymes, moving from the lowest to the highest SUA levels (*P* < 0.05).

**Table 1:** Baseline characteristics of participants according to uric acid classification (n = 1451)

	** Serum Uric acid (mg/dl)**		
	**≤5** **(n=965)**	**>5** **(n=486)**	***P*** ** value **
**Gender n(%)**			< 0.001^€^
Male	329(34.1)	396(81.5)	
Female	636(65.9)	90(18.5)	
**Education level n(%)**			0.226^€^
≤ diploma	237 (24.6)	134(27.6)	
University	728(75.4)	352(72.4)	
**Marital status n(%)**			0.001^€^
Not married	135(14)	39(8)	
Married	830(86)	447(92)	
**Cardiometabolic phenotype n (%)**			< 0.001^€^
^*^MHL	257(26.6)	61(12.6)	
^**^MUHL	9(0.9)	3(0.6)	
^¶^MHO	586(60.7)	320(65.8)	
^¶^MUHO	113(11.7)	102(21)	
**Smoking status n(%)**			0.066^€^
Yes	52(5.4)	39(8.1)	
	**mean±SD**	**mean±SD**	
Age (year)	42.34 ± 6.59	42.91 ± 6.96	0.134^¥^
Weight (kg)	73.41 ± 12.27	83.06 ± 13.44	< 0.001^¥^
Waist circumference (cm)	94.02 ± 8.74	98.12 ± 9.21	< 0.001^¥^
Body mass index (kg/m^2^)	27.39 ± 4.01	28.56 ± 3.79	< 0.001^¥^
Triglyceride (mg/dl) *Median (interquartile rang)*	107.26 ± 48.72 95(48)	149.40 ± 74.44 130(80.25)	< 0.001^¥¥^
Fasting blood sugar (mg/dl) *Median (interquartile rang)*	85.26 ± 21.08 82 (14)	88.58 ± 18.16 86(13.25)	< 0.001^¥¥^
Blood urea nitrogen (mg/dl)	12.09 ± 3.24	13.32 ± 3.16	< 0.001^¥^
Creatinine (mg/dl)	0.97 ± 0.14	1.10 ± 0.16	< 0.001^¥^
High density lipoprotein (mg/dl)	47.52 ± 10.85	42.64 ± 9.11	< 0.001^¥^
Cholesterol (mg/dl)	163.02 ± 35.66	176.63 ± 37.61	< 0.001^¥^
Low density lipoprotein (mg/dl)	94.18 ± 29.52	104.56 ± 30.24	< 0.001^¥^
Systolic blood pressure (mm Hg)	108.30 ± 14.30	115.83 ± 13.55	< 0.001^¥^
Diastolic blood pressure (mm Hg)	74.64 ± 9.07	79.19 ± 8.83	< 0.001^¥^
Aspartate aminotransferase (IU/l) *Median (interquartile rang)*	18.37 ± 6.89 17(6.50)	23.72 ± 10.32 21(9)	< 0.001^¥¥^
Alanin amino transferase (IU/l) *Median (interquartile rang)*	20.62 ± 12.32 17(12)	33.58 ± 21.63 28(21)	< 0.001^¥¥^
Alkaline phosphatase (IU/l)	168.85 ± 49.75	188.59 ± 53.33	< 0.001^¥^
Gamma glutamyle transferase (IU/l) *Median (interquartile rang)*	20.10 ± 16.48 16(11)	29.76 ± 18.04 25(18)	< 0.001^¥¥^

^€^*P* value: chi square test;^¥^*P*- value: Independent t test; ^¥¥^*P* value MannWhitney U test; *MHL: metabolically healthy lean; ^**^MUHL: metabolically unhealthy lean; ^¶^MHO: Metabolically healthy obese; ^¶¶^MUHO: metabolically unhealthy obese

Table 2 shows that in all cardiometabolic phenotype classes, the lowest proportion of females (*P* < 0.001) was in the highest SUA category.

**Table 2:** Demographic, anthropometric and biochemical factors according to serum uric acid classification stratified by cardiometabolic phenotype

	**Serum Uric acid level (mg/dl)**		
	**≤5** **(n=965)**	**>5** **(n=486))**	***P*** ** value**
*MHL			
Gender n(%)			^€^ < 0.001
Male	109(42.4)	55(90.2)	
Female	148(57.6)	6(9.8)	
	**mean±SD**	**mean±SD**	
Age (year)	41.30 ± 6.65	41.11 ± 7.48	^€^0.849
Weight (kg)	63.50 ± 8.47	67.86 ± 7.18	^¥^ < 0.001
Waist circumference (cm)	86.07 ± 5.81	86.66 ± 5.53	^¥^0.472
Triglyceride (mg/dl) *Median (interquartile rang)*	93.47 ± 39.41 84(38)	113.311 ± 41.45 107(64)	^¥¥^ < 0.001
Fasting blood sugar (mg/dl) *Median (interquartile rang)*	82.69 ± 13.48 81(12)	83.24 ± 8.54 83(12.50)	^¥¥^0.159
Blood urea nitrogen (mg/dl)	12.10 ± 3.34	13.04 ± 2.87	^¥^0.04
Creatinine (mg/dl)	0.98 ± 0.14	1.11 ± 0.17	^¥^ < 0.001
High density lipoprotein (mg/dl)	48.87 ± 10.89	44.30 ± 8.77	^¥^0.002
Cholesterol (mg/dl)	156.75 ± 34.91	166.24 ± 28.76	^¥^0.050
Low density lipoprotein (mg/dl)	89.17 ± 28.75	99.29 ± 23.99	^¥^0.011
Aspartate aminotransferase (IU/l) *Median (interquartile rang)*	17.67 ± 6.22 16(6)	21.49 ± 10.12 20(6)	^¥^ < 0.001
Alanin amino transferase (IU/l) *Median (interquartile rang)*	18.29 ± 10.44 16(10)	26.52 ± 15.48 23(17)	^¥¥^ < 0.001
Alkaline phosphatase (IU/l)	161.69 ± 43.16	182.96 ± 48.24	^¥^0.001
Gamma glutamyle transferase (IU/l) *Median (interquartile rang)*	16.74 ± 9.58 14(8)	21.81 ± 8.52 20(12)	^¥¥^ < 0.001
Systolic blood pressure (mm Hg)	104.19 ± 12.72	109.95 ± 12.38	^¥^0.002
Diastolic blood pressure (mm Hg)	71.85 ± 7.52	74.83 ± 7.28	^¥^0.006
**MUHL			
Gender n(%)			^€^0.01
Male	2(22.2)	3(100)	
Female	7(77.8)	0	
	**mean±SD**	**mean±SD**	
Age (year)	44.67 ± 6.38	43.67 ± 7.57	^¥^0.826
Weight (kg)	66.81 ± 9.61	70.96 ± 4.66	^¥^0.497
Waist circumference (cm)	92.87 ± 3.73	85.23 ± 3.30	^¥^0.011
Triglyceride (mg/dl) *Median (interquartile rang)*	164.77 ± 84.69 161(93.5)	215.33 ± 41.45 226	^¥¥^0.162
Fasting blood sugar (mg/dl) *Median (interquartile rang)*	87.44 ± 31.67 75(13)	90 ± 14.79 97	^¥¥^0.401
Blood urea nitrogen (mg/dl)	12.88 ± 4.07	12.66 ± 2.30	^¥^0.932
Creatinine (mg/dl)	0.88 ± 0.12	1.03 ± 0.24	^¥^0.191
High density lipoprotein (mg/dl)	40.78 ± 5.09	41.00 ± 7.93	^¥^0.950
Cholesterol (mg/dl)	165.11 ± 31.74	163.33 ± 44.06	^¥^0.940
Low density lipoprotein (mg/dl)	91.33 ± 28.90	79.33 ± 40.67	^¥^0.582
Aspartate aminotransferase (IU/l) *Median (interquartile rang)*	26 ± 30.47 16(5.50)	23 ± 6.24 21	^¥¥^0.113
Alanin amino transferase (IU/l) *Median (interquartile rang)*	28.55 ± 35.12 15(11.5)	30.33 ± 19.62 19	^¥¥^0.305
Alkaline phosphatase (IU/l)	152.44 ± 42.32	190.66 ± 62.42	^¥^0.251
Gamma glutamyle transferase (IU/l) *Median (interquartile rang)*	26.77 ± 33.08 15(11.5)	20 ± 2.0 20	^¥¥^0.353
Systolic blood pressure (mm Hg)	107.96 ± 11.53	135.33 ± 6.02	^¥^0.003
Diastolic blood pressure (mm Hg)	75.59 ± 13.20	87.55 ± 7.04	^¥^0.173
***¶MHO ***			
Gender n(%)			^€^ < 0.001
Male	174(29.7)	267(83.4)	
Female	412(70.3)	53(16.6)	
	**mean±SD**	**mean±SD**	
Age (year)	42.26 ± 6.37	43.05 ± 7.01	^¥^0.080
Weight (kg)	75.98 ± 10.70	84.21 ± 11.82	^¥^ < 0.001
Waist circumference (cm)	95.99 ± 7.38	98.54 ± 8.14	^¥^ < 0.001
Triglyceride (mg/dl) *Median (interquartile rang)*	101.62 ± 36.23 94(39.25)	137.35 ± 64.81 122(64.25)	^¥¥^ < 0.001
Fasting blood sugar (mg/dl) *Median (interquartile rang)*	82.76 ± 15.71 81(13)	86.23 ± 10.53 85(13)	^¥¥^ < 0.001
Blood urea nitrogen (mg/dl)	12.02 ± 3.18	13.65 ± 3.23	^¥^ < 0.001
Creatinine (mg/dl)	0.96 ± 0.14	1.11 ± 0.17	^¥^ < 0.001
High density lipoprotein (mg/dl)	48.23 ± 10.89	43.49 ± 9.15	^¥^ < 0.001
Cholesterol (mg/dl)	164.40 ± 35.29	176.51 ± 38.18	^¥^ < 0.001
Low density lipoprotein (mg/dl)	95.83 ± 29.16	105.88 ± 31.07	^¥^ < 0.001
Aspartate aminotransferase (IU/l) Median (interquartile rang)	18.31 ± 6.07 17(6.25)	23.55 ± 9.70 21.5(9.75)	^¥¥^ < 0.001
Alanin amino transferase (IU/l) Median (interquartile rang)	20.67 ± 11.73 17(11)	33.20 ± 20.64 28(19.75)	^¥¥^ < 0.001
Alkaline phosphatase (IU/l)	170.34 ± 51.55	188.95 ± 51.25	^¥^ < 0.001
Gamma glutamyle transferase (IU/l) Median (interquartile rang)	20.32 ± 17.89 16(11)	30.13 ± 18.78 26(17.75)	^¥¥^ < 0.001
Systolic blood pressure (mm Hg)	108.09 ± 13.95	114.65 ± 12.50	^¥^ < 0.001
Diastolic blood pressure (mm Hg)	74.71 ± 9.09	78.49 ± 8.0	^¥^ < 0.001
***¶¶MUHO***			
Gender n(%)	44(38.9)	71(69.6)	^€^ < 0.001
Male	69(61.1)	31(30.4)	
Female			
	**mean±SD**	**mean±SD**	
Age (year)	44.98 ± 6.89	43.50 ± 6.36	^¥^0.104
Weight (kg)	83.12 ± 12.99	88.92 ± 14.57	^¥^0.002
Waist circumference (cm)	101.94 ± 8.10	104.05 ± 7.69	^¥^0.052
Triglyceride (mg/dl) *Median (interquartile rang)*	163.30 ± 74.0 156(80.50)	206.88 ± 87.02 191(94.75)	^¥¥^ < 0.001
Fasting blood sugar (mg/dl) *Median (interquartile rang)*	103.93 ± 40.60 92(26)	99.09 ± 32.25 90(21)	^¥¥^0.52
Blood urea nitrogen (mg/dl)	12.33 ± 3.30	12.46 ± 3.01	^¥^0.749
Creatinine (mg/dl)	0.99 ± 0.16	1.09 ± 0.16	^¥^ < 0.001
High density lipoprotein (mg/dl)	41.30 ± 8.50	39.01 ± 8.37	^¥^0.048
Cholesterol (mg/dl)	169.96 ± 37.84	183.59 ± 39.23	^¥^0.010
Low density lipoprotein (mg/dl)	97.20 ± 32.13	104.30 ± 30.41	^¥^0.098
Aspartate aminotransferase (IU/l) *Median (interquartile rang)*	19.62 ± 7.29 18(9)	25.60 ± 12.06 23(12.50)	^¥¥^ < 0.001
Alanin amino transferase (IU/l) *Median (interquartile rang)*	25 ± 14.60 21(17)	39.07 ± 26.25 32(27.25)	^¥¥^ < 0.001
Alkaline phosphatase (IU/l)	178.68 ± 52.78	190.77 ± 62.26	^¥^0.125
Gamma glutamyle transferase (IU/l) *Median (interquartile rang)*	26.09 ± 17.71 22(14.50)	33.64 ± 18.66 29(19.25)	^¥¥^ < 0.001
Systolic blood pressure (mm Hg)	118.83 ± 14.58	122.40 ± 14.69	^¥^0.076
Diastolic blood pressure (mm Hg)	80.60 ± 9.04	83.70 ± 8.83	^¥^0.012

€*P* value: chi square test; ¥ *P*- value: Independent t test; ¥¥ *P* value: Mannwhitney U test; * MHL: metabolically healthy lean; ** MUHL: metabolically unhealthy lean; ¶MHO: Metabolically healthy obese; ¶¶MUHO: metabolically unhealthy obese

 The average values for serum TG, Cr, AST, ALT, and GGT in the MHL, MHO, and MUHO groups had an increasing dose-response trend according to the SUA classification (*P* < 0.05). The SBP and DBP values also increased significantly in a similar trend.

 In contrast to the other factors, WC, and cholesterol did not rise with increasing SUA. In addition, WC decreased noticeably in the MUHL group based on SUA tertiles (*P* = 0.01). Table 3 presents the relationship between the SUA level and the cardiometabolic phenotype. The multinomial regression analysis indicates that compared to the lowest SUA category, the OR of becoming MHO and MUHO increased by 2.30 (95%CI: 1.68–3.13) and 3.80 (95%CI: 2.58–5.59), respectively. After adjustment for different confounding factors, the correlation was still significant. The highest OR for becoming MHO (OR 3.13;2.12-4.44) and MUHO (5.50;3.53-8.57) was observed in the third model. In contrast, no similar trend was observed for the relationship between MUHL and the SUA tertile.

**Table 3:** Odds ratios and 95% confidence intervals for cardiometabolic phenotype according to serum uric acid classification

**Serum uric acid levels**		
	**>5 mg/dl**	***P*** ** value **
	OR(95% CI)	
^*^ **MUHL**		
Unadjusted	1.40(0.36-5.34)	0.618
Model 1	1.96(0.45-8.53)	0.370
Model2	1.94(0.42-8.87)	0.389
^**^ **MHO **		
Unadjusted	2.30(1.68-3.13)	< 0.001
Model1	3.01(2.13-4.24)	< 0.001
Model2	3.13(2.21-4.44)	< 0.001
^***^ **MUHO**		
Unadjusted	3.80(2.58-5.59)	< 0.001
Model 1	5.15(3.32-7.98)	< 0.001
Model2	5.50(3.53-8.57)	< 0.001

* MUHL: metabolically unhealthy lean; **MHO: Metabolically healthy obese; ***MUHO: metabolically unhealthy obeseMHLgroup (metabolically healthy lean) was considered as a reference group; Model 1: adjusted for age and gender; Model 2 adjusted for age, gender; education level,smoking status

## Discussion

 We assessed the relationship between serum uric acid (SUA) and the cardiometabolic phenotype, revealing a dose-dependent rise in the mean values of metabolic syndrome (MetS) measures, including LDL and cholesterol, with increased SUA levels. These results are similar to the findings of previous studies in various countries.^11,18,19^ According to our findings, the prevalence rates of MHO and MUHL were higher in the second category than the first. Compared to the lowest SUA category, the odds of MHO and MUHO increased by 3.13 (95% CI 2.21–4.44) and 5.50 (95%CI 3.53–8.57) in the highest category, respectively.

 To our knowledge, the relationship between SUA and the cardiometabolic phenotype has not previously been examined. Therefore, we contrasted our results with previous studies evaluating the connection between SUA and MetS. An increase in MetS due to the elevation of SUA levels has been reported.^20^ The current study’s results align with the findings of Ishizaka et al who evaluated the relationship between SUA and MetS among participants with BMI ≥ 25 kg/m^2^. They noticed that in the highest SUA quartile, the odds of MetS increased by 2.27% (95% CI: 1.90–2.72) after adjusting for the confounding factors. ^21^ In our study, after adjusting for the confounding factors, the risk of MUHO at the highest level was more than that of the study by Ishizaka et al^21^ (OR 5.50 vs 2.27). This variation is potentially due to the differences in defining MetS, where they considered a BMI > 25 kg/m^2^ as a marker for MetS, while we determined MetS according to ATP III, and then categorized the participants based on BMI (BMI < 25 or ≥ 25 kg/m^2^).

 The relationship between SUA homeostasis and MetS is highly complex.^22^ It remains debatable whether an elevated SUA level is a risk factor or just a biomarker for the progress of MetS. ^23^ Some researchers have stated that hyperuricemia can be an exclusive component of MetS,^24,25^ while others put forth hyperuricemia as a supplementary component of MetS.^23,26^ Elevated SUA levels will cause outcomes such as hypertension,^27^ hypertriglyceridemia, and low high density lipoprotein.^28^ Several pathways have been suggested to explain the link between SUA and MetS. First is endothelial dysfunction, proven to be caused by hyperuricemia in human and animal subjects.^29,30^ Secondly, uric acid prevents nitric oxide (NO) production,^31^ which is involved in the accurate functioning of insulin.^32^ Therefore, hyperuricemia may play a potential role in causing and increasing insulin resistance. Insulin resistance is known to play an essential role in the pathogenesis of MetS.^33^ Thirdly, another role of uric acid involves inducing oxidative stress, which causes inflammation in adipocytes^34,35^ and hepatocytes.^36^ However, the complicated correlation between uric acid and oxidative stress is noteworthy because it can be paradoxical.^37^ In other words, while uric acid is an antioxidant that disables superoxide anion, peroxynitrite, and hydroxyl radical, there is some evidence showing that under ischemic stress or high SUA, uric acid functions as a pro-oxidant.^38^

 In our study, we noticed that the presence of males in the highest SUA level was significantly higher than their presence in the lowest category. In line with our results, this phenomenon has been noted in previous studies.^39,40^ The lower tubular urate post-secretory reabsorption and the higher renal clearance of urate in women may explain this observation.^41^

 According to our findings, the average serum levels of liver enzymes were elevated in MHL, MHO, and MUHO individuals in a dose-response manner corresponding to SUA classification. Interestingly, the mean serum liver enzyme levels increased with SUA levels in MHL individuals. This may suggest a BMI-independent association between liver enzymes and SUA. At the same time, it may indicate that the MHL subjects in the highest category are at risk of becoming MUHL. Nevertheless, we did not observe the same trend in the MUHL group, which might be due to the limited sample size of this group. These findings are in line with the findings of prior studies.^42,43^ For instance, Shih et al state that individuals with hyperuricemia are more likely to have heightened liver enzymes (AST or ALT), even after adjusting for confounders.^42^ It has been reported non-alcoholic fatty liver disease (NAFLD) is closely linked with obesity, diabetes mellitus, and MetS.^44-46^ Therefore, NAFLD is believed to be a hepatic outcome of metabolic diseases.^47^ It turns out that SUA levels increase in most NAFLD patients,^48^ indicating that it can be an independent predisposing factor for NAFLD.^49^

 The current study’s main limitation is that due to its cross-sectional design, causal inferences regarding the relationship between SUA and the cardiometabolic phenotype could not be evaluated. However, the main strength is that it is the first to evaluate the relationship between SUA and the cardiometabolic phenotype. SUA is easily accessible in regular clinical practice and is measured using standardized techniques. It would be useful to distinguish the transition from MHO to MUHO since it may lead to earlier and more precise identification of MHO subjects at risk of transition to MUHL, which can facilitate the administration of better preventive strategies. Another strength of this work lies in using data from a large cohort study.

## Conclusion

 Our findings indicate that increases in the prevalence of MHO and MUHO are related to elevated SUA levels. Furthermore, the average values for the components of MetS and the lipid profile increased with the elevation in SUA levels. Additionally, there was a positive dose-response relationship concerning the mean levels of serum liver enzymes in the MHL, MHO, and MUHO groups. Accordingly, we propose using the easily-measured SUA level as a marker for the early diagnosis of at-risk MUHL and MHO individuals in order to provide proper interventions. However, the mechanisms that cause SUA to lead to this disorder remain elusive. Consequently, further prospective works are needed to identify the effects of SUA on the progression of MetS in various body-size subgroups.

## Competing Interests

 The authors declare that they have no competing interests

## Ethical Approval

 This study was approved by the ethics committee of Tabriz University of Medical Sciences (IR.TBZMED.REC.1400.657).
